# Excitonic Complexes in n-Doped WS_2_ Monolayer

**DOI:** 10.1021/acs.nanolett.0c05021

**Published:** 2021-03-08

**Authors:** Małgorzata Zinkiewicz, Tomasz Woźniak, Tomasz Kazimierczuk, Piotr Kapuscinski, Kacper Oreszczuk, Magdalena Grzeszczyk, Miroslav Bartoš, Karol Nogajewski, Kenji Watanabe, Takashi Taniguchi, Clement Faugeras, Piotr Kossacki, Marek Potemski, Adam Babiński, Maciej R. Molas

**Affiliations:** † Institute of Experimental Physics, Faculty of Physics, University of Warsaw, ul. Pasteura 5, 02-093 Warsaw, Poland; ‡ Department of Semiconductor Materials Engineering, Wrocław University of Science and Technology, Wybrzeże Wyspiańskiego 27, 50-370 Wrocław, Poland; § Laboratoire National des Champs Magnétiques Intenses, CNRS-UGA-UPS-INSA-EMFL, 25, avenue des Martyrs, 38042 Grenoble, France; ∥ Department of Experimental Physics, Wrocław University of Science and Technology, Wybrzeze Wyspianskiego 27, 50-370 Wrocław, Poland; ⊥ Central European Institute of Technology, Brno University of Technology, Purkyňova 656/123, 612 00 Brno, Czech Republic; # Research Center for Functional Materials, National Institute for Materials Science, 1-1 Namiki, Tsukuba 305-0044, Japan; ∇ International Center for Materials Nanoarchitectonics, National Institute for Materials Science, 1-1 Namiki, Tsukuba 305-0044, Japan

**Keywords:** tungsten disulfide monolayer, exciton, trion, dark exciton, phonon replica, biexciton

## Abstract

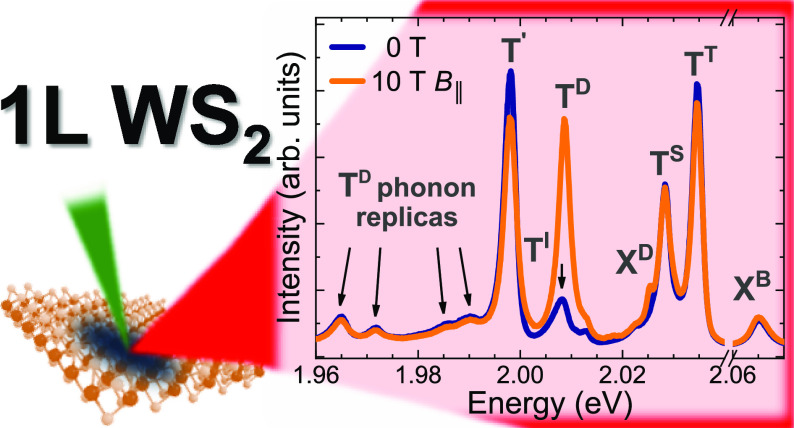

We investigate the origin of emission lines apparent in the low-temperature photoluminescence spectra of n-doped WS_2_ monolayer embedded in hexagonal BN layers using external magnetic fields and first-principles calculations. Apart from the neutral A exciton line, all observed emission lines are related to the negatively charged excitons. Consequently, we identify emissions due to both the bright (singlet and triplet) and dark (spin- and momentum-forbidden) negative trions as well as the phonon replicas of the latter optically inactive complexes. The semidark trions and negative biexcitons are distinguished. On the basis of their experimentally extracted and theoretically calculated *g*-factors, we identify three distinct families of emissions due to exciton complexes in WS_2_: bright, intravalley, and intervalley dark. The *g*-factors of the spin-split subbands in both the conduction and valence bands are also determined.

Monolayers (MLs) of semiconducting transition metal dichalcogenides (S-TMDs) MX_2_, where M = Mo or W and X = S, Se, or Te, are direct band gap semiconductors with the minima of the conduction band (CB) and maxima of the valence band (VB) located at the inequivalent K^±^ points of their hexagonal Brillouin zone (BZ).^1,2^ The strong spin–orbit interaction and lack of inversion symmetry result in the splitting of the VB (Δ_*v*_) and the CB (Δ_*c*_) extrema. Whereas the former splitting is of the order of a few hundreds of millielectronvolts, the latter equals only a few tens of millielectronvolts and can be positive or negative.^3^ Consequently, two subgroups of MLs can be distinguished: “bright” (the excitonic ground state is optically active or bright) comprising MoSe_2_ and MoTe_2_,^1,4,5^ and “darkish” (the excitonic ground state is optically inactive or dark) composed of MoS_2_, WS_2_, and WSe_2_.^4−6^

Dark excitons in S-TMD ML can be divided into two subgroups because of the distinct origins of their optical inactivity, that is, intravalley spin-forbidden and intervalley momentum-forbidden complexes, which can not recombine optically due to the spin and momentum conservation rule for excitons. Dark excitonic complexes can be also characterized by their net charge. Both neutral and charged dark excitons exist, which are bound electron–hole (e–h) pairs and the bound e–h pairs with an extra carrier (an electron or a hole), respectively.

In this work, we investigate the low-temperature optical response of high-quality n-doped tungsten disulfide (WS_2_) ML encapsulated hexagonal BN (hBN) flakes using photoluminescence (PL) spectroscopy in external magnetic fields. All emission lines, observed in the PL spectrum, are due to both the bright (singlet and triplet) and dark (spin- and momentum-forbidden) negative trions as well as the phonon replicas of the latter optically inactive complexes. Moreover, the semidark trions and negative biexcitons are distinguished. Magneto-PL measurements accompanied by first-principles calculations allow us to extract the *g*-factors of all transitions as well as of the spin-split subbands in both the conduction and the valence bands.

The negatively charged exciton (negative trion) is a three-particle complex composed of an e–h pair and an excess electron. There are four negative trions in W-based darkish MLs (WS_2_ or WSe_2_), that is, two bright and two dark states, see Figure 1. These states can be formed in both the K^+^ and K^–^ valleys (taking into account the location of a hole), which leads to two possible configurations of a given complex. Because of the spin conservation rule for S-TMD MLs, the bright (optically active) negative trion may be found in both the intravalley singlet (T^S^) and intervalley triplet (T^T^) states. They involve correspondingly two electrons from the same valley whereas the triplet trion comprises two electrons from different valleys. For the dark (optically inactive) negative trions, the corresponding electrons are located in different valleys and are characterized by the antiparallel alignment of their spins. This configuration leads to two complexes, depending on the electron involved in the recombination process: intravalley spin- (T^D^) and intervalley momentum-forbidden (T^I^), which cannot recombine optically because of the spin and momentum conservation, respectively.

**Figure 1:**
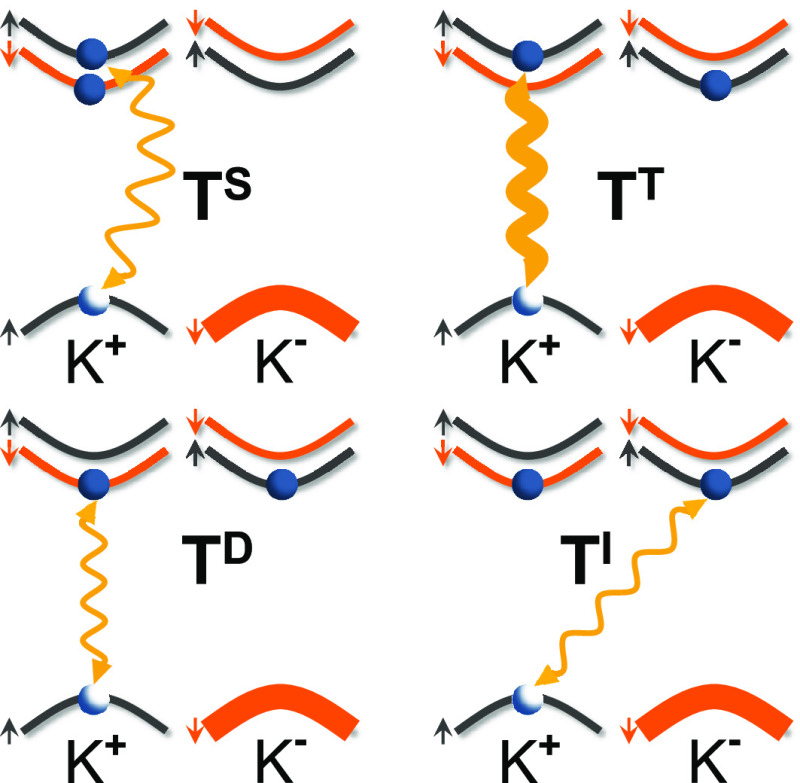
Schematic illustration of possible spin configurations for negatively charged excitons formed in the vicinity of so-called A exciton. T^S^ and T^T^ correspond to the bright singlet and triplet trions, whereas T^D^ and T^I^ represent the dark intravalley and intervalley complexes, respectively. Note that we draw only complexes for which a hole is located at the K^+^ point of the BZ.

In order to investigate the negatively charged complexes in the WS_2_ ML, we measured its low temperature (*T* = 5 K) PL spectrum at zero magnetic field and in the in-plane magnetic field of 10 T, see Figure 2. It is well established that the application of an in-plane magnetic field (*B*_∥_) results in the mixing of the bright and dark excitons which becomes apparent in the optical activation of spin-forbidden dark complexes.^4−10^ The zero-field PL spectrum is composed of several emission lines. On the basis of the previous reports,^4,11−15^ three peaks can be assigned unquestionably to the bright neutral exciton (X^B^) and to two bright negatively charged excitons, that is, singlet (T^S^) and triplet (T^T^), formed in the vicinity of the optical band gap (A exciton). Two additional lines, labeled X^D/G^ and T^D^ become apparent in *B*_∥_ = 10 T (see Figure 2). As it was recently reported,^10^ the X^D/G^ peak corresponded to the dark and gray states of the neutral exciton, whereas the T^D^ peak was related to the dark state of the negative trion. The PL spectra, shown in Figure 2, also comprise several lines at lower energies, which are denoted as T^I^, T′, T_ZA(K)_^D^, T_LA(K)_^D^, T_E″(K)_^D^, and T_E″(Γ)_^D^. Increasing the excitation power leads to the appearance of an additional emission, labeled XX^–^. Those lines have not been reported so far in WS_2_ MLs and the following is dedicated to their identification.

**Figure 2:**
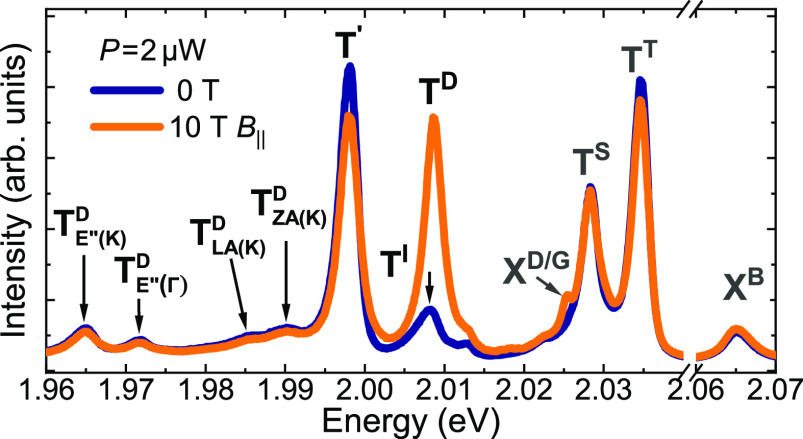
Low-temperature PL spectra measured on an n-doped WS_2_ ML encapsulated in hBN flakes at zero magnetic field and in the in-plane magnetic field of *B*_∥_ = 10 T.

As can be appreciated in Figure 1, both the intravalley spin-forbidden and intervalley momentum-forbidden negative trions share the same initial carrier configuration. The difference arises from their recombination pathway. Whereas the T^D^ complex involves recombination of an e–h pair from the same K^±^ point, for the T^I^ trion an electron and a hole from neighboring K^±^ valleys recombine. The T^D^ can be identified with magnetic brightening experiments with a *B*_∥_ field, see Figure 3a. This effect was investigated in details in ref (10). The observation and assignment of the emission to the intervalley momentum-forbidden negative trion is more striking. Recently, similar emission related to the momentum-forbidden dark neutral exciton was reported in the WSe_2_ ML.^16,17^ It was demonstrated that at zero magnetic field the intensity of the momentum-forbidden emission was much smaller as compared to the corresponding spin-forbidden one. In our case, the T^I^ line dominates at *B* = 0 T, whereas the T^D^ one can be only observed because of the brightening effect. As the energy difference between those complexes is only of about 530 μeV, the optical emission of the T^I^ line through the Auger processes^18^ or by emission of optical phonons can be excluded.^16^ Similar to the case of the indirect band gap in thin layers of WS_2_,^19^ the optical recombination of intervalley dark trion can be allowed due to defect states, which may provide momentum conservation during recombination. The origin of the T^D^-T^I^ energy splitting is not clear as both the intervalley and intravalley dark trions share the same carrier configuration (see Figure 1). We believe that this splitting arises from higher-order processes; the description of this is beyond the scope of our work. The similar energy separation between the intervalley and intravalley dark neutral excitons in WSe_2_ ML was reported to be of about 10 meV and was ascribed to a short-range electron–hole exchange interaction.^16,17^ Note that to exclude the assignment of the T^I^ line to the recently reported Q–K-valley momentum-indirect excitons,^20,21^ we performed additional analysis in Supporting Information (SI).

**Figure 3:**
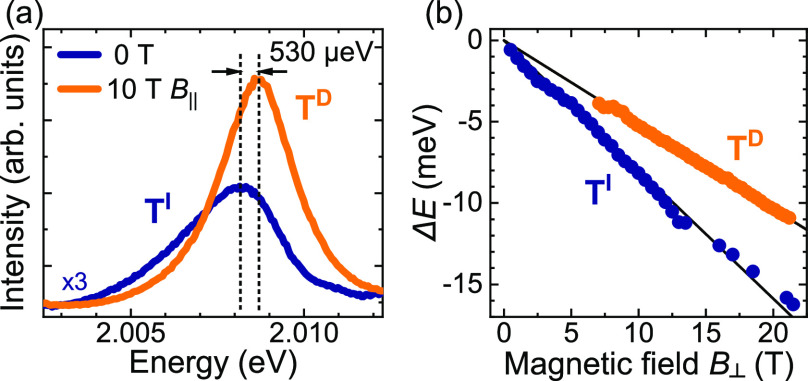
(a) Low-temperature emission due to the intravalley T^D^ and intervalley T^I^ dark trions measured on an n-doped WS_2_ ML encapsulated in hBN flakes at zero magnetic field and in the in-plane magnetic field of *B*_∥_ = 10 T. (b) Energy separation between the two circularly polarized split components of the T^D^ and T^I^ transitions as a function of the out-of-plane magnetic field *B*_⊥_. The solid lines represent fits according to the equation described in the text. Note the measurements were performed in the tilted configuration of the magnetic field direction in respect to the ML plane (see Supporting Information for details).

Upon application of an out-of-plane magnetic field (*B*_⊥_), excitonic transitions split into two circularly polarized components (σ^±^). Their energy separation Δ*E*(*B*) = *E*_σ+_ – *E*_σ-_ can be expressed as Δ*E*(*B*) = *gμ*_B_*B*_⊥_, where *g* denotes the *g*-factor of the considered excitonic complex and μ_B_ is the Bohr magneton. The magnetic field evolution of the Δ*E* with linear fits to experimental data for both the T^D^ and T^I^ excitons is shown in Figure 3b. Linear fits to the experimental data are also presented in the figure. The resulting *g*-factors of the T^D^ and T^I^ are equal to −8.9 and −13.7, respectively. The former value is consistent with our recent result reported in ref (10), whereas the latter one is very similar to the *g*-factor reported for intervalley dark complexes in WSe_2_ MLs.^16,17^

The identification of four lines apparent in the lowest energy range of the PL spectrum (see Figure 2) will be addressed in the following. One of the possibilities to fulfill the spin and momentum conservation during optical recombination of the dark trions is phonon emission. Figure 4a shows a schematic illustration of possible recombination pathways of dark negative trions involving phonon emission. The phonon-assisted processes give rise to so-called phonon replicas of dark excitons in WSe_2_ MLs.^16,17,22−25^ Because of the symmetry and the momentum of a given phonon, it may lead to the transfer of a carrier (an electron or a hole) to a virtual state with a spin-flip or a valley-flip.

**Figure 4:**
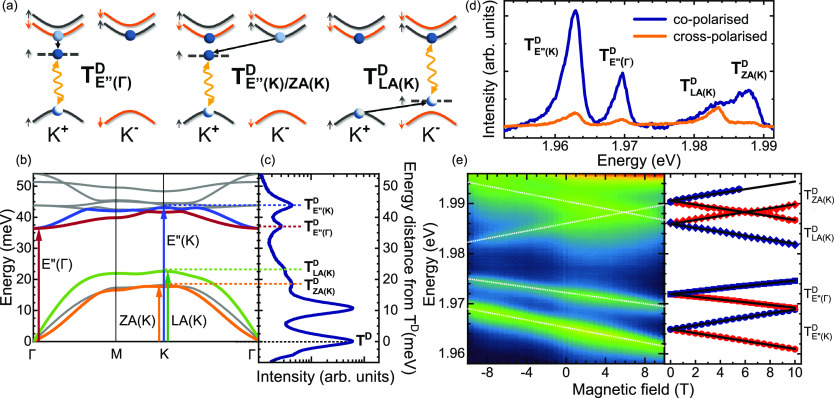
(a) Schematic illustration of possible recombination pathways of dark trions assisted by the emission of optical (E″) and acoustic (ZA, LA) phonons from the K or Γ points of the BZ, which give rise to the PL of so-called phonon replicas. The black solid lines represent the phonon emission, which transfer an electron or a hole from the real subband in the CB or VB to the virtual state denoted by a dashed horizontal line. Only complexes for which a hole is located at the K^+^ point of the BZ are drawn. (b) The calculated phonon dispersion of WS_2_ ML. The dispersions of the pertinent phonon modes are indicated by color curves, whereas for the others it is represented by gray curves. (c) Low-temperature PL spectrum due to the intravalley T^D^ dark trion and its phonon replicas measured on the studied WS_2_ ML at *B*_∥_ = 10 T. Note that the energy axis in panel (c) is relative, that is, in reference to the T^D^ emission. (d) Helicity-resolved low-temperature PL spectrum with the emission lines related to phonon replicas of the studied ML at zero magnetic field under circularly polarized excitation with the T^S^ energy. (e) (left panel) False-color PL map as a function of out-of-plane magnetic field (*B*_⊥_). Note that the positive and negative values of magnetic fields correspond to σ^±^ polarizations of detection. White dashed lines superimposed on the observed transitions are guides to the eyes. (right panel) Transition energies of the σ^±^ (red/blue points) components of the studied line related to phonon replicas as a function of the out-of-plane magnetic field. The solid lines represent fits according to the equation described in the text.

The phonon replicas of the dark trion should be red-shifted from it by the phonon energies with the redshift corresponding to the phonon emission. The calculated phonon dispersion can be therefore compared to the low-temperature PL spectrum of the WS_2_ ML as presented in Figure 4b,c. We found that the extracted relative energies of phonon replicas from the T^D^ line are in good agreement with the corresponding theoretical phonon energies. To confirm the assignment of phonons shown in Figure 4a, we analyzed their symmetries following the group theory considerations and irreducible representations (IR) notation from ref (26), where WSe_2_ ML of the same symmetry as WS_2_ ML was studied. The intravalley spin-flip process of an electron can only be assisted by a phonon, which transforms like the IR Γ_5_, that is, E″(Γ). Additionally, comparing the measured redshift (37 meV) and the calculated phonon energy (36.4 meV), the phonon replica observed at about 1.97 eV can be identified as T_E″(Γ)_^D^. The other replicas, which involve the momentum-flip processes (the spin of the electrons is conserved at the same time), must be induced by phonons from the K point. It is possible to transfer an electron (hole) between K^±^ valleys with emission of E″(K) (LA(K)) phonons, as they transform according to IR K_3_ (K_1_). Their calculated energies (43.0 and 22.6 meV) agree well with the measured redshifts (44 and 23 meV). Therefore, we label the replicas apparent at about 1.965 and 1.986 eV as T_LA(K)_^D^ and T_E″(K)_^D^. The red shift of the replica at around 1.99 eV (18 meV) is close to calculated energies of TA(K) and ZA(K) phonons (18.2 and 17.7 meV). The former one should induce a spin flip, whereas the latter one preserves this symmetry and couples to a spin conserving transition. Because of the extracted *g*-factor value for this replica (discussed in the next paragraph), we label it as T_ZA(K)_^D^.

To confirm the assignment of the phonon replicas, we analyze their polarization properties under circular polarized excitation and their evolution when applying an out-of-plane magnetic field. Figure 4d shows the helicity-resolved PL under circularly polarized excitation with the T^S^ energy. After formation of the T^S^ complex at the K^±^ point using the σ^±^ polarization, an electron from the top subband of the CB at K^±^ point relaxes to the bottom one at the opposite K^∓^ point, which leads to the formation of the dark trion (see Figure 1). As a result of the spin- and momentum-flip processes of electrons in the CB, three replicas (T_E″(Γ)_^D^, T_E″(K)_^D^, T_ZA(K)_^D^) are characterized by large conservation of excitation helicity in emission. For the T_LA(K)_^D^ line, the opposite behavior should be present, as the emission occurs at the valley which is opposite to the excitation one due to the momentum-flip of a hole in the VB. However, that replica demonstrates almost zero preservation of excitation helicity, which may be related to the scattering processes of carriers.^27^ These polarization properties also affect the PL spectra measured in *B*_⊥_ field. As can be seen in Figure 4e, the studied lines are characterized not only by different magnitudes of their field-induced shifts but also by the sign. To extract their *g*-factors, we fitted our experimental results using the formula 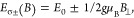
where *E*_0_ is the emission energy at zero field. The obtained *g*-factors of −13.6, −13.3, and +13.6 for T_E″(K)_^D^, T_ZA(K)_^D^, and T_LA(K)_^D^, respectively, are consistent with the T^I^ one (−13.7). Note that the sign of the T_LA(K)_^D^*g*-factor is the opposite, which is an indication of the intervalley transfer of hole, whereas the *g*-factor of T_E″(Γ)_^D^ of −8.9 is identical to the value obtained for T^D^.

One of the most pronounced features observed in the PL spectrum is the T′ line in which the intensity is comparable to the T^S^ and T^T^ ones, see Figure 2. Although the emission line was previously observed many times in the low-temperature PL spectra of both the WSe_2_ and WS_2_ MLs,^14,16,17,22−24,28,29^ its origin is not well-defined. We ascribe the T′ line to the recombination of the dark trion made optically active due to the electron–electron (e–e) scattering,^30^ and name it a semidark trion. We note that the corresponding semidark trion has recently been reported in WSe_2_ ML.^31^ The initial state of the T′ line is dark, the same as for both the T^D^ and T^I^ trions; compare Figures 5a and 1. Because of the intervalley e–e scattering, an electron located at the lower CB subband in the K^±^ point is transferred to the higher lying CB subband in the opposite K∓ valley, which results in the optical recombination of an e–h pair.

**Figure 5:**
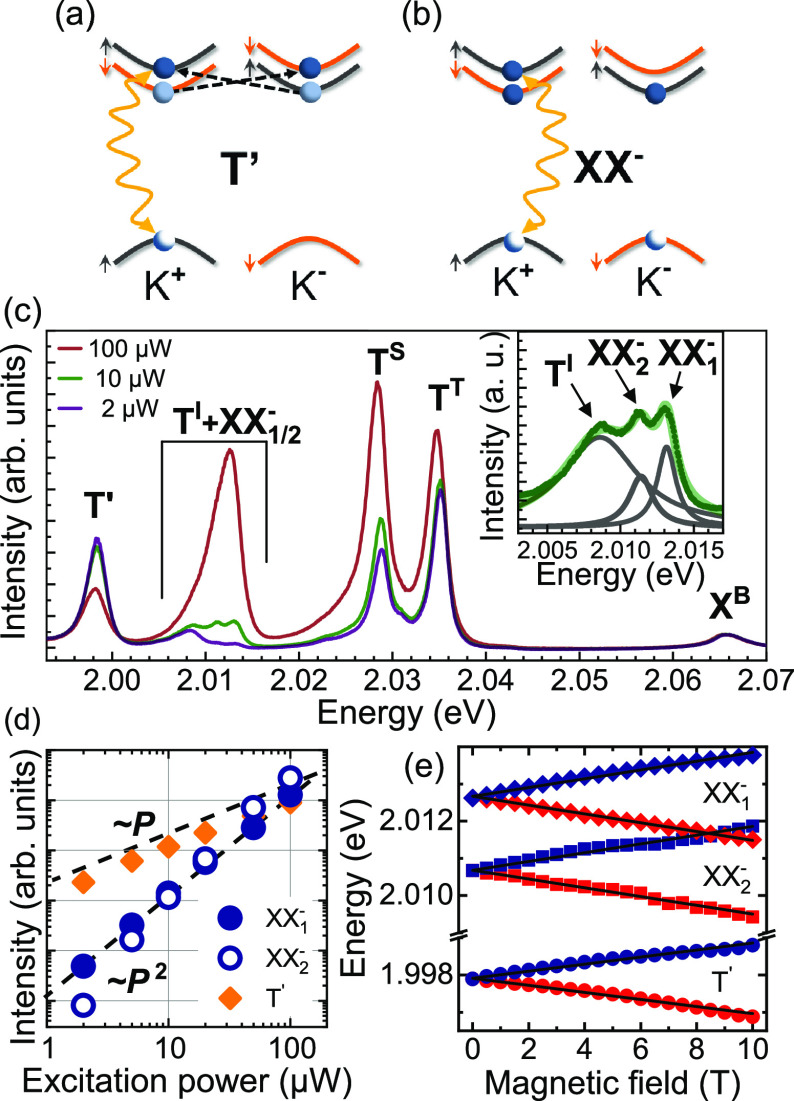
Schematic illustration of a possible recombination pathway of (a) semidark trions made optically active due to the e–e scattering and (b) negative biexcitons. The black dashed lines represent e–e scattering, which transfers an electron from the lower subband of the CB at the K^±^ point to the corresponding higher subband at the opposite K^∓^ point. (c) Power dependence of the low-temperature PL spectra of the WS_2_ ML. The intensities of the PL spectra are normalized by the X^B^ intensity. The inset displays the PL spectrum under 10 μW excitation due to the T^I^, XX_1_^–^, and XX_2_^–^ lines deconvoluted using Lorentzian function. (d) The intensity evolution of the emission features with excitation power. The dashed black line indicates the linear and quadratic behaviors as a guide to the eye. (e) Transition energies of the σ^±^ (red/blue points) components of the T′, XX_1_^–^, and XX_2_^–^ lines as a function of the out-of-plane magnetic field. The solid lines represent fits according to the equation described in the text.

The last studied excitonic complex is a negative biexciton, denoted as XX^–^. Its formation is possible due to the long lifetime of dark trions (see Figure 5b), which was reported to be close to 0.5 ns.^10^ The emission related to negative biexcitons is only apparent at high excitation power and it is characterized by two lines, labeled XX_1_^–^ and XX_2_^–^, see Figure 5c. The XX_1_^–^–XX_2_^–^ energy separation of about 2 meV is very similar to the energy separation reported for two neutral biexcitons (2.5 meV) in WSe_2_ ML.^32^ Surprisingly, as two lines of the neutral biexcitons can be explained in terms of two possible carrier configurations, the appearance of the XX_1_^–^ and XX_2_^–^ lines is not clear (there is a single possible configuration, see Figure 5b) which requires more sophisticated theoretical analysis.

To confirm our assignment of the T′, XX_1_^–^, and XX_2_^–^ lines, Figure 5d presents their intensity evolution as a function of excitation power. We found that T′ emission follows a linear behavior, whereas the XX_1_^–^ and XX_2_^–^ peaks are characterized by superlinear evolution. These types of power dependence are typical for excitonic complexes composed of a single e–h pair or by two e–h pairs.^32−34^ Note that the excitation-power evolution of other excitons is presented in SI. Additionally, we analyze their evolution in the *B*_⊥_ fields, see Figure 5e. Using the same approach as for phonon replicas, we extracted *g*-factors of −3.3, −4.1, and −4.1 for T′, XX_1_^–^, and XX_2_^–^ lines, respectively. These values are consistent with the *g*-factors found for the bright complexes, such as X^B^, T^S^, T^T^ (see SI for details). The additional analysis of the T′ line is presented in SI.

The *g*-factors for the all studied excitonic complexes are summarized in Table 1. According to the extracted values of *g*-factors, the excitonic complexes can be arranged into three groups: (i) *g*-factors in the range −3.3 to −4.1 are characteristic for bright transitions (X^B^, T^S^, T^T^, T′, XX_1_^–^, and XX_2_^–^); (ii) the spin-forbidden dark transitions are described by the *g*-factor equal to −8.9 (T^D^ and T_E″(Γ)_^D^); and (iii) values of *g*-factors of about −13.3 to −13.7 and +13.6 are obtained for momentum-forbidden dark transitions (T^I^, T_E″(K)_^D^, T_ZA(K)_^D^, and T_LA(K)_^D^). In order to establish the *g*-factors of single subbands in both the CB and VB, we adapted the method proposed in ref (25). It relies on the comparison of *g*-factors related to the different excitonic complexes (see SI for details). The obtained values of the *g*-factors for higher-energy *c* + 1 (*v*) and lower-energy *c* (*v* – 1) subbands in CB (VB) at the K^+^ point are shown in Table 2. Note that the *g*-factor of the *v* – 1 band was calculated using the reported *g*-factor of B exciton (−4) in ref (35). The extracted values demonstrate that the simple model commonly employed for the calculation of the excitonic *g*-factors using additive contribution of the spin, valley, and orbital angular momenta^36^ cannot explain the single band *g*-factors.

**Table 1:** Experimental (*g*^exp^) and (*g*^calc^) *g*-Factors of Investigated Emission Lines^a^

	*g*^exp^	*g*^calc^	Δ*L*	ΔΣ	*P*
X^B^	–3.5	–3.56	–1.78	0	σ+
T^S^	–4.0				
T^T^	–3.9				
T′	–3.3				
XX_1_^–^	–4.1				
XX_2_^–^	–4.1				
					
T^D^	–8.9	–8.73	–2.37	–2	σ+
T_E″(Γ)_^D^	–8.9				
					
T^I^	–13.7	–12.20	–6.10	0	σ+
T_E″(K)_^D^	–13.6				
T_ZA(K)_^D^	–13.3				
					
T_LA(K)_^D^	+13.6	+12.20	–6.10	0	σ–

a Δ*L* and ΔΣ represent orbital and spin contributions to *g*_calc_ at K^+^, respectively. The helicity of the given transition occurring at K^+^ valley, denoted as *P*, is shown in the last column.

**Table 2:** Experimental (*g*_*n*_^exp^) and theoretical (*g*_*n*_^calc^) *g*-factors, orbital (L_*n*_) and spin (Σ_*n*_) angular momenta of valence (*n*: *v* −1, *v*) and conduction (*n*: *c*, *c* + 1) bands at K^+^ point

*n*	*g*_*n*_^exp^	*g*_*n*_^calc^	L_*n*_	Σ_*n*_
*v* – 1	2.81	2.79	3.79	–1
*v*	6.10	5.23	4.23	+1
*c*	0.86	0.87	1.87	–1
*c* + 1	3.84	3.45	2.45	+1

To verify our experimental results, we calculate theoretically *g*-factors using a first-principles based approach proposed in ref (37). In this case, first the *g*-factors of single subbands (*g*_*n*_^calc^) are calculated, which are then used to determine the *g*-factor of a given transition (*g*^calc^). Consequently, the theoretical *g*-factor of the band *n* (*n* = *v* – 1, *v*, *c*, *c* + 1) at point K^+^ is evaluated as *g*_*n*,K+_^calc^=*L*_*n*,K+_ + Σ_*n*,K+_, where *L*_*n*,K+_ and Σ_*n*,K+_ are the orbital and spin angular momenta of the bands hosting the bound e–h pair involved in the optical recombination process, whereas the excess carriers do not contribute. A scissor correction to the experimental free-particle gap was applied during the evaluation of *L*_*n*,K+_.^38^ The *g*-factors of studied excitons can then be expressed as *g*^calc^ = ±2(*g*_*c*(+1),K+_^calc^ – *g*_*v*(−1),K+_^calc^) = ± 2(Δ*L*_K+_ + ΔΣ_K+_), where Δ denotes the difference of respective angular momenta between the *c*(+1) and *v*(−1) bands. The sign is determined by the polarization of the transition at K^+^ valley, which reflects the optical selection rules. We obtain three values of *g*^calc^: −3.56, −8.73, and ±12.20, which correspond to bright, spin-forbidden, and momentum-forbidden transitions groups, respectively. The magnitudes of *g*-factors can be explained in terms of the orbital and spin contributions to the angular momenta of bands. The bright complexes involve the spin-conserving transitions, therefore their *g*^calc^ is determined only by the orbital contribution. For the spin-forbidden complexes, the change of spin part is equal to −2 and the orbital part is also enhanced, as band *c* is involved in the transition. The momentum-forbidden complexes involve carriers from different K valleys, which give rise to the large orbital angular momentum difference and lead to a high value of *g*^calc^. For T_LA(K)_^D^ the *g*-factor is positive because K^–^ valley couples to σ^–^ light. As can be appreciated in Table 2, the experimental and theoretical values of *g*-factors are in very good agreement. The spread of experimentally obtained *g*-factors from −3.3 to −4.1 for the bright group cannot be explained with the employed approach and requires further theoretical investigations.

Note that the presented low-temperature PL spectrum of WS_2_ ML is very similar to the reported ones for WSe_2_,^8,16,17,22−24,28,29,32−34^ whereas both of them are completely different from the MoS_2_ spectra probably due to the reversed and small (about 3 meV) CB spin–orbit splitting of the latter material.

We identified all emission lines apparent in the low-temperature PL spectra of n-doped WS_2_ ML embedded in hBN layers using external magnetic fields. We found that the extracted *g*-factors of all transitions may be arranged in three groups revealing a nature of electron–hole recombination: bright, intravalley, and intervalley dark. We explained their signs and magnitudes with the aid of first-principles calculations. This division can open an opportunity to identify the origin of the reported so-called localized excitons in the emission spectra of the WSe_2_ and WS_2_ MLs exfoliated on Si/SiO_2_ substrates. The obtained *g*-factors of the spin-split subbands in both the CB and VB are important for better understanding of the interlayer transitions in van der Waals heterostructures.

## Supporting Information Available

Sample, experimental setups, theoretical calculations, Q–K-valley momentum indirect excitons, excitation power evolution of excitonic emissions, suppression/saturation of the T′ line, *g*-factors of excitonic complexes, conduction, and valence bands *g*-factors (PDF)
