# Correction to “Excitonic Complexes in n-Doped WS_2_ Monolayer”

**DOI:** 10.1021/acs.nanolett.1c03616

**Published:** 2021-10-18

**Authors:** Małgorzata Zinkiewicz, Tomasz Woźniak, Tomasz Kazimierczuk, Piotr Kapuscinski, Kacper Oreszczuk, Magdalena Grzeszczyk, Miroslav Bartoš, Karol Nogajewski, Kenji Watanabe, Takashi Taniguchi, Clement Faugeras, Piotr Kossacki, Marek Potemski, Adam Babiński, Maciej R. Molas

On March 8th, 2021, the manuscript entitled “Excitonic Complexes in n-Doped WS_2_ Monolayer” by M. Zinkiewicz et al. was published as a Letter in *Nano Letters*.

Numerical values placed in the column *g*_*n*_^exp^ in Table 2 are not our experimentally obtained values as was expected. The valid numerical values are corrected in Table 2 below.

**Table 2:** Experimental (*g*_*n*_^exp^) and theoretical (*g*_*n*_^calc^) *g*-factors, orbital (L_*n*_) and spin (Σ_*n*_) angular momenta of valence (*n*: *v* – 1, *v*) and conduction (*n*: *c*, *c* + 1) bands at K^+^ point

*n*	*g*_*n*_^exp^	*g*_*n*_^calc^	L_*n*_	Σ_*n*_
*v* – 1	3.08	2.79	3.79	–1
*v*	5.47	5.23	4.23	+1
*c*	1.08	0.87	1.87	–1
*c* + 1	3.70	3.45	2.45	+1

