# Supplementary material for: Excitonic Complexes in n-Doped WS2 Monolayer
Source: Nano Lett. 2021 Mar 8;21(6):2519–25. doi: 10.1021/acs.nanolett.0c05021 (PMC7995249; doi:10.1021/acs.nanolett.0c05021)
Supplement: Supplementary file 1 — nl0c05021_si_001.pdf [file nl0c05021_si_001.pdf]

Supplemental Information: Excitonic complexes in n-doped WS_2 monolayer

Małgorzata Zinkiewicz,[1,*] Tomasz Woźniak,[2] Tomasz Kazimierczuk,[1] Piotr Kapuscinski,[3,4] Kacper Oreszczuk,[1] Magdalena Grzeszczyk,[1] Miroslav Bartoš,[3,5] Karol Nogajewski,[1] Kenji Watanabe,[6] Takashi Taniguchi,[7] Clement Faugeras,[3] Piotr Kossacki,[1] Marek Potemski,[1,3] Adam Babiński,[1] and Maciej R. Molas[1,†]

[1]*Institute of Experimental Physics, Faculty of Physics, University of Warsaw, ul. Pasteura 5, 02-093 Warsaw, Poland*
[2]*Department of Semiconductor Materials Engineering, Wrocław University of Science and Technology, Wybrzeże Wyspiańskiego 27, 50-370 Wrocław, Poland*
[3]*Laboratoire National des Champs Magnétiques Intenses, CNRS-UGA-UPS-INSA-EMFL, 25, avenue des Martyrs, 38042 Grenoble, France*
[4]*Department of Experimental Physics, Wrocław University of Science and Technology, ul. Wybrzeże Wyspiańskiego 27, 50-370 Wrocław, Poland*
[5]*Central European Institute of Technology, Brno University of Technology, Purkyňova 656/123, 612 00 Brno, Czech Republic*
[6]*Research Center for Functional Materials, National Institute for Materials Science, 1-1 Namiki, Tsukuba 305-0044, Japan*
[7]*International Center for Materials Nanoarchitectonics, National Institute for Materials Science, 1-1 Namiki, Tsukuba 305-0044, Japan*

S1. SAMPLE

The studied sample is composed of WS_2 ML encapsulated in hBN flakes and supported by a bare Si substrate. The structure was obtained by two-stage polydimethylsiloxane (PDMS)-based[1] mechanical exfoliation of WS_2 and hBN bulk crystals. A bottom layer of hBN in the hBN/WS_2/hBN heterostructure was created in the course of a non-deterministic exfoliation. The assembly of the hBN/WS_2/hBN heterostructure was realized via successive dry transfers of WS_2 ML and capping hBN flake from PDMS stamps onto the bottom hBN layer.

S2. EXPERIMENTAL SETUPS

Low-temperature micro-magneto-PL experiments were performed in the Voigt, Faraday and tilted geometries, *i.e.* magnetic field oriented parallel, perpendicular, and 45° with respect to ML's plane. Measurements (spatial resolution $\sim$2 μm) were carried out with the aid of two systems: a split-coil superconducting magnet and a resistive solenoid producing fields up to 10 T and 30 T using a free-beam-optics arrangement and an optical-fiber-based insert, respectively. The sample was placed on top of a x-y-z piezo-stage kept at T=10 K or T=4.2 K and was excited using a laser diode with 532 nm or 515 nm wavelength (2.33 eV or 2.41 eV photon energy). The emitted light was dispersed with a 0.5 m long monochromator and detected with a charge coupled device (CCD) camera. For measurements up to 10 T, the circular polarizations of the emissions were analyzed using a set of polarizers and a $\lambda/4$ waveplate placed directly in front of the spectrometer. In tilted-field configurations in high magnetic fields, the combination of a quarter wave plate and a linear polarizer placed in the insert were used to analyse the circular polarization of signals (the measurements were performed with a fixed circular polarization, whereas reversing the direction of magnetic field yields the information corresponding to the other polarization component due to time-reversal symmetry). Note that the excitation power for experiments performed in magnetic fields up to 30 T was adjusted based on the comparison of the measured PL spectrum and the one obtained under excitation of laser with 532 nm.

S3. THEORETICAL CALCULATIONS

First principles calculations were performed with the use of *Vienna Ab Initio Simulation Package* (VASP)[2] and the Projector Augmented Wave method[3]. After testing several exchange-correlation functionals, van der Waals corrections and functionals, the parametrization of Perdew-Burke-Ernzerhof revised for solids (PBEsol)[4] was used for geometry optimization, as yielding the best agreement with experimental lattice constant and layer thickness[5]. Atomic positions and lattice constants were optimized with 10^{-5} eV/Å and 0.1 kbar precision. Energy cutoff of 400 eV, a $12 \times 12 \times 1$ k-mesh and 15 Å of vacuum in the vertical direction of unit cell were chosen after careful convergence tests. Phonon band structure calculations were performed using Phonopy package[6], which implements the finite displacement method to obtain the interatomic force constants[7]. A $3 \times 3 \times 1$ supercell was found sufficient to yield converged phonon energies at Γ and K points. The orbital angular momenta of bands were obtained from the wave function derivatives that are calculated within density functional perturbation theory[8].

S4. Q-K-VALLEY MOMENTUM-INDIRECT EXCITONS

The issue of indirect-momentum excitons formed by a hole in K valley and an electron in Q valley recently reported for ML WS_2[9] and WSe_2[10] should also be addressed. For WS_2 ML, the energy separation between the bright excitons (X^B) at K point and the Q-K-valley momentum-indirect excitons was reported in the range of 70 to 100 meV[11,12]. This suggests that the contribution of the latter one to our measured low-temperature PL spectra may be apparent. We evaluate the magnitude of g-factors of the Q-K-valley momentum-indirect excitons to confirm the validity of our assignment of the excitonic lines. From our calculations the spin and orbital angular momenta of the $Q^{\pm}$ valley in CB are ±1 and ±0.006. As the precise selection rules for indirect-momentum excitons are not yet known, we can estimate the absolute value of Q-K excitons to be $|g| \approx |2g_v^{K^{\pm}}| = 8.46$, assuming spin-direct excitons. Surprisingly the calculated g-factor value is nearly the same as the extracted experimentally absolute value of the g-factor for the spin-forbidden dark trion (T^D) (8.9). However, the identification of the T^D is straightforward, as the brightening effect of the in-plane magnetic field is well established. The resulting mixing of the bright and dark excitons, becomes apparent in the optical activation of spin-forbidden dark complexes[13–19]. The phonon replica line ($T^D_{E''(\Gamma)}$) is characterized by the g-factor of 8.9, but its assignment is evident because of the same value of its g-factor as of T^D and of the T^D-$T^D_{E''(\Gamma)}$ energy separation matches nicely to the E"(Γ) phonon energy. For the T^I trion and the $T^D_{E''(K)}$, $T^D_{ZA(K)}$, and $T^D_{LA(K)}$ phonon replicas, their extracted experimentally absolute values of the g-factors (13.3-13.7) vary significantly from the theoretical value for Q-K-valley momentum-indirect excitons. The analogous approach can be applied for other lines, *i.e.* X^B, T^S, T^T, T', XX_1^-, and XX_2^-, distinguished by the absolute values of g-factors from 3.3 to 4.1. Consequently, the attribution of the different investigated emission lines to the recombination of Q-K indirect excitons must be excluded.

S5. EXCITATION POWER EVOLUTIONS OF EXCITONIC EMISSIONS

In order to verify the assignment of different investigated excitonic complexes, we measured excitation power dependency of the PL spectra. Fig. S1 demonstrates the integrated intensity of excitons as a function of excitation powers. As can be appreciated, most of the complexes, *i.e.* X^B, T^T, T^S, T^I, T', $T^D_{E''(\Gamma)}$, and $T^D_{E''(K)}$, are characterized by nearly linear dependence, while the intensity growth of both the XX_1^- and XX_2^- lines are described by superlinear evolution. These types of power dependences are typical for excitonic complexes composed of a single electron-hole (e-h) pair or by two e-h pairs[20–22].

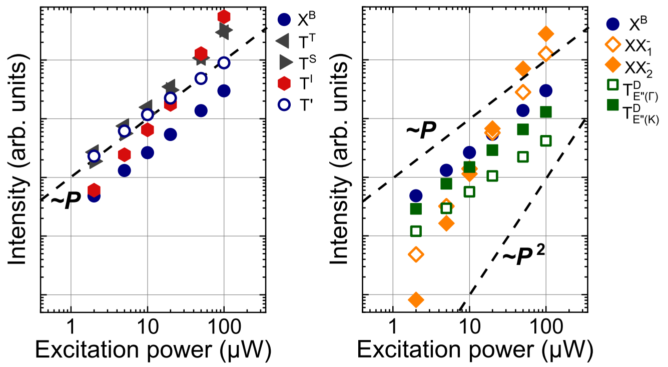

FIG. S1. The intensity evolution of the emission features with excitation power in the log-log plot. The dashed black line indicates the linear and quadratic behaviours as a guide to the eye.

S6. g-FACTORS OF EXCITONIC COMPLEXES

Fig. S2(a) illustrates the false-color map of the magneto-PL spectra measured in magnetic fields up to 10 T oriented perpendicular to ML's plane under power excitation of 20 μW, which allows us to investigate the g-factors of negatively charged biexcitons (XX_1^- and XX_2^-). Upon application of an out-of-plane magnetic field, the excitonic emissions split into two circularly polarized components due to the excitonic Zeeman effect[23]. Their energies evolutions ($E(B)$) in

external out-of-plane magnetic fields ($B_\perp$) can be described as:

$$E(B) = E_0 \pm \frac{1}{2} g \mu_\mathrm{B} B_\perp, \tag{1}$$

where E_0 is the energy of the transition at zero field, g denotes the g-factor of the considered excitonic complex and μ_B is the Bohr magneton. The fitting results of Eq. 1 to the experimental results are presented in Fig. S2(b). As can be seen, for all the excitonic complexes, the observed evolutions can be described by the aforementioned formula. We found that the obtained values of g-factors could be organized in three groups: ∼4 (black fitted lines), ∼9 (navy fitted lines), and ∼13 (orange fitted lines). The g-factors value for each excitonic complex as well as the origin of three groups are presented in the main article.

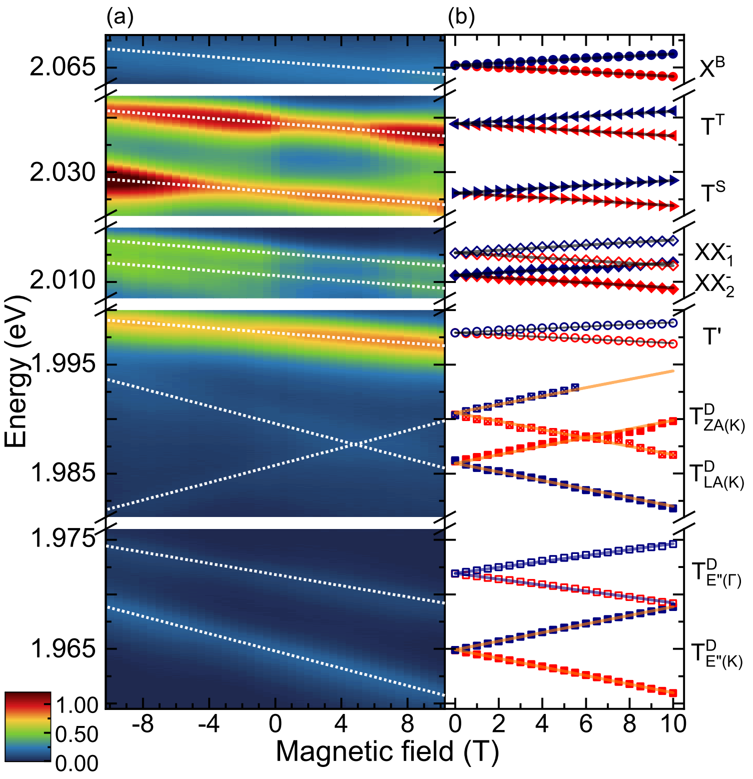

FIG. S2. (a) False-color map of the PL response as a function of $B_\perp$. Note that the positive and negative values of magnetic fields correspond to $\sigma^\pm$ polarizations of detection. The intensity scale is logarithmic. White dashed lines superimposed on the investigated transitions are guides to the eyes. (b) Transition energies of the $\sigma^{+/-}$ (red/blue points) components of different excitonic complexes lines as a function of the out-of-plane magnetic field. The solid lines represent fits according to Eq. 1. Three groups of g-factors with absolute values close to 4, 9 and 13 are indicated with different colours black, navy and orange respectively.

S7. SUPPRESSION/SATURATION OF THE T' LINE

As the suppression/saturation of the T' line is observed in Fig. 2 and 5(c) in the main text, we decided to analyse these effects.

The initial state of the spin- ($\mathrm{T^D}$) and momentum-forbidden ($\mathrm{T^I}$) dark excitons and also semi-dark trion (T') is the same [compare Figs. 1 and 5(a) in the main text]. For Fig. 2, there are two presented PL spectra: zero-field and measured in in-plane magnetic field of 10 T. It is well established that the application of the in-plane magnetic field leads to the brightening effect of the spin-forbidden dark excitons in S-TMD MLs, *i.e.* radiative recombination of these types of complexes. In our case, it manifests in the observation of the emission line due to the $\mathrm{T^D}$ trion. As a result, the population of the initial state for the $\mathrm{T^D}$, $\mathrm{T^I}$ and T' trions decrease significantly, which causes the suppression of the T' emission line. Similar situation takes place for measurements performed as a function of excitation power, shown in Fig. 5(c) in the main text. In that case, however, the interplay takes place between the T' and $\mathrm{XX^-}$

complexes. The final state of the negative biexciton and the initial state of the semi-dark trion are the same [compare Figs. 5(a) and (b) in the main text]. As the intensities of the XX^- lines increase superlinearly as a function of excitation power, the formation of the negative biexcitons is favoured at the highest excitation powers, while the T' intensity starts to saturate. These two effects suggest that the T' complex cannot recombine radiatively, because the formation of the XX^- complexes is much faster.

To summarize, the observed suppression/saturation of the T' line is consistent with assignment of this complex to the semi-dark trion.

S8. CONDUCTION AND VALENCE BANDS g-FACTORS

Recently, a method for determination of single subbands g-factors in the WSe_2 MLs was proposed in Ref. 24. As low temperature PL spectra of both the WSe_2 and WS_2 MLs comprise of several emission lines including so-called phonon replicas, we have adapted this method to obtain g-factors of single conduction and valence subbands in WS_2 ML. Within this approach, we compare energy evolution of spin-forbidden dark trion ($\mathrm{T^D}$) and its phonon replica ($\mathrm{T^D_{E"(K)}}$) in out-of-plane magnetic fields ($B_\perp$). Particularly, the energy difference between their circularly-polarized components ($\sigma^\pm$) corresponding to emissions from different valleys ($\mathrm{K^+}$ or $\mathrm{K^-}$) is analysed, see Fig. S3(a). Fig. S3(b) shows the magnetic field evolution of the $\sigma^\pm$ components of the $\mathrm{T^D}$ and $\mathrm{T^D_{E"(K)}}$ lines. Note that the field dependence of $\mathrm{T^D}$ emission was measured in the tilted configuration of the sample by 45° with respect to the applied magnetic field direction, which gives both in-plane and out-of-plane components of magnetic fields, see Ref. 19 for details. There are four energy distances marked in Fig. S3(b) by black vertical lines: $\Delta\sigma^+$, $\Delta\sigma^-$, Δmin and Δmax. With help of schematic illustration shown in Fig. S3(a), it follows that they depend only on single subband g-factors and phonon energy and are equal to:

$$
\begin{aligned}
\Delta\sigma^+ &= \mathrm{T^D(K^+)} - \mathrm{T^D_{E"(K)}(K^+)} = \mathrm{E"(K)} + 2g_c\mu_B B_\perp,\\
\Delta\sigma^- &= \mathrm{T^D(K^-)} - \mathrm{T^D_{E"(K)}(K^-)} = \mathrm{E"(K)} - 2g_c\mu_B B_\perp,\\
\Delta min &= \mathrm{T^D(K^+)} - \mathrm{T^D_{E"(K)}(K^-)} = \mathrm{E"(K)} - 2g_v\mu_B B_\perp,\\
\Delta max &= \mathrm{T^D(K^-)} - \mathrm{T^D_{E"(K)}(K^+)} = \mathrm{E"(K)} + 2g_v\mu_B B_\perp,
\end{aligned} \tag{2}
$$

where $\mathrm{T^D(K^\pm)}$ and $\mathrm{T^D_{E"(K)}(K^\pm)}$ represent the magnetic field evolutions of $\sigma^\pm$ components of spin forbidden dark trion ($\mathrm{T^D}$) and its E" phonon replica ($\mathrm{T^D_{E"(K)}}$). E"(K) is energy of E" phonon at K point. g_c and g_v are the g-factors of the lowest conduction band g_c and the highest valence band g_v, while μ_B and $B_\perp$ are related to the Bohr's magneton and the applied out-of-plane magnetic field.

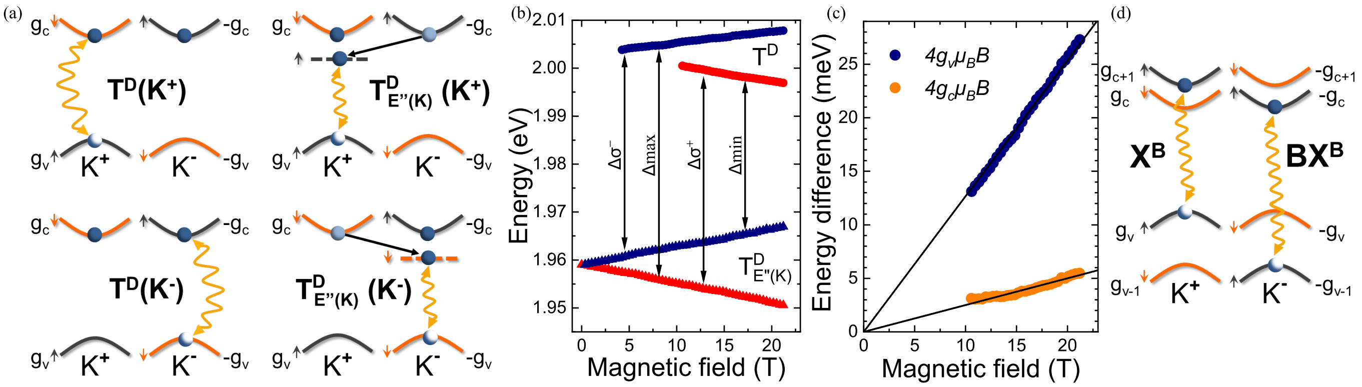

FIG. S3. (a) Schematic illustration of possible configurations for spin-forbidden negative dark trion ($\mathrm{T^D}$) negatively charged dark exciton and its phonons replica ($\mathrm{T^D_{E"(K)}}$) for both valleys denoted as $\mathrm{K^+}$ and $\mathrm{K^-}$ in the bracket. (b) Transition energies of the $\sigma^{+/-}$ (red/blue points) components of the $\mathrm{T^D}$ and $\mathrm{T^D_{E"(K)}}$ transitions as a function of the out-of-plane magnetic field. Black arrows show energy differences between optical transition marked as Δmin, Δmax, $\Delta\sigma^+$ and $\Delta\sigma^-$. (c) Magnetic field evolution of energy differences described by Eq. 3. Black solid lines represent linear fits to the designated points. (d) Schematic illustration of possible configurations for the bright A ($\mathrm{X^B}$) and B ($\mathrm{BX^B}$) excitons formed in the $\mathrm{K^+}$ and $\mathrm{K^-}$ points, respectively.

Further transformations of the aforementioned equations lead to establishing equations which allow determination of g_c and g_v values:

$$4g_c\mu_B B = \Delta\sigma^- - \Delta\sigma^+,$$
$$4g_v\mu_B B = \Delta max - \Delta min, \tag{3}$$

Fig. S3(c) demonstrates experimentally obtained evolutions with linear fits marked with solid black lines. Obtained values are equal to 1.1 and 5.5 for g_c and g_v, respectively. That is in a good agreement with theoretical calculations, which are discussed widely in the main article.

Knowing values of g_c and g_v, we are able to determine the g-factor of top conduction band subband (g_{c+1}) from previously obtained g-factor of the bright A exciton, *i.e.* $\mathrm{X^B}$ line, with the aid of formula: $g_{\mathrm{X^B}} = 2(g_{c+1} - g_v)$. We extracted that g_{c+1} value is equal to 3.7.

Finally, taking g-factor of the bright B exciton ($g_{\mathrm{BX^B}}$) from Ref. 25, it is possible to determine the g-factor of the bottom valence subband (g_{v-1}) using formula: $g_{\mathrm{BX^B}} = 2(g_c - g_{v-1})$. We found that g_{v-1} value is equal to 2.9.

---

* malgorzata.zinkiewicz@fuw.edu.pl
† maciej.molas@fuw.edu.pl

[1] A. Castellanos-Gomez, M. Buscema, R. Molenaar, V. Singh, L. Janssen, H. S. J. van der Zant, and G. A. Steele, 2D Materials **1**, 011002 (2014).
[2] G. Kresse and J. Furthmüller, Physical Review B **54**, 11169 (1996).
[3] G. Kresse and D. Joubert, Physical Review B **59**, 1758 (1999).
[4] J. P. Perdew, A. Ruzsinszky, G. I. Csonka, O. A. Vydrov, G. E. Scuseria, L. A. Constantin, X. Zhou, and K. Burke, Physical Review Letters **100**, 136406 (2008).
[5] W. Schutte, J. De Boer, and F. Jellinek, Journal of Solid State Chemistry **70**, 207 (1987).
[6] A. Togo and I. Tanaka, Scripta Materialia **108**, 1 (2015).
[7] K. Parlinski, Z. Q. Li, and Y. Kawazoe, Physical Review Letters **78**, 4063 (1997).
[8] M. Gajdoš, K. Hummer, G. Kresse, J. Furthmüller, and F. Bechstedt, Physical Review B **73**, 045112 (2006).
[9] D. Bao, A. G. del Águila, T. Thu Ha Do, S. Liu, J. Pei, and Q. Xiong, 2D Materials **7**, 031002 (2020).
[10] J. Madéo, M. K. L. Man, C. Sahoo, M. Campbell, V. Pareek, E. L. Wong, A. Al-Mahboob, N. S. Chan, A. Karmakar, B. M. K. Mariserla, X. Li, T. F. Heinz, T. Cao, and K. M. Dani, Science **370**, 1199 (2020).
[11] M. Selig, G. Berghäuser, A. Raja, P. Nagler, C. Schüller, T. F. Heinz, T. Korn, A. Chernikov, E. Malic, and A. Knorr, Nature Communications **7**, 13279 (2016).
[12] E. Malic, M. Selig, M. Feierabend, S. Brem, D. Christiansen, F. Wendler, A. Knorr, and G. Berghäuser, Physical Review Materials **2**, 014002 (2018).
[13] A. O. Slobodeniuk and D. M. Basko, 2D Materials **3**, 035009 (2016).
[14] M. R. Molas, C. Faugeras, A. O. Slobodeniuk, K. Nogajewski, M. Bartos, D. M. Basko, and M. Potemski, 2D Materials **4**, 021003 (2017).
[15] X.-X. Zhang, T. Cao, Z. Lu, Y.-C. Lin, F. Zhang, Y. Wang, Z. Li, J. C. Hone, J. A. Robinson, D. Smirnov, S. G. Louie, and T. F. Heinz, Nature Nanotechnology **12**, 883 (2017).
[16] M. R. Molas, A. O. Slobodeniuk, T. Kazimierczuk, K. Nogajewski, M. Bartos, P. Kapuściński, K. Oreszczuk, K. Watanabe, T. Taniguchi, C. Faugeras, P. Kossacki, D. M. Basko, and M. Potemski, Physical Review Letters **123**, 096803 (2019).
[17] Z. Lu, D. Rhodes, Z. Li, D. V. Tuan, Y. Jiang, J. Ludwig, Z. Jiang, Z. Lian, S.-F. Shi, J. Hone, H. Dery, and D. Smirnov, 2D Materials **7**, 015017 (2019).
[18] C. Robert, B. Han, P. Kapuscinski, A. Delhomme, C. Faugeras, T. Amand, M. R. Molas, M. Bartos, K. Watanabe, T. Taniguchi, B. Urbaszek, M. Potemski, and X. Marie, Nature Communications **11**, 4037 (2020).
[19] M. Zinkiewicz, A. O. Slobodeniuk, T. Kazimierczuk, P. Kapuściński, K. Oreszczuk, M. Grzeszczyk, M. Bartos, K. Nogajewski, K. Watanabe, T. Taniguchi, C. Faugeras, P. Kossacki, M. Potemski, A. Babiński, and M. R. Molas, Nanoscale **12**, 18153 (2020).
[20] M. Barbone, A. R. P. Montblanch, D. M. Kara, C. Palacios-Berraquero, A. R. Cadore, D. De Fazio, B. Pingault, E. Mostaani, H. Li, B. Chen, K. Watanabe, T. Taniguchi, S. Tongay, G. Wang, A. C. Ferrari, and M. Atatüre, Nature Communications **9**, 3721 (2018).
[21] S.-Y. Chen, T. Goldstein, T. Taniguchi, K. Watanabe, and J. Yan, Nature Communications **9**, 3717 (2018).
[22] Z. Li, T. Wang, Z. Lu, C. Jin, Y. Chen, Y. Meng, Z. Lian, T. Taniguchi, K. Watanabe, S. Zhang, D. Smirnov, and S.-F. Shi, Nature Communications **9**, 3719 (2018).
[23] M. Koperski, M. R. Molas, A. Arora, K. Nogajewski, M. Bartos, J. Wyzula, D. Vaclavkova, P. Kossacki, and M. Potemski, 2D Materials **6**, 015001 (2019).
[24] C. Robert, H. Dery, L. Ren, D. van Tuan, E. Courtade, M. Yang, B. Urbaszek, D. Lagarde, K. Watanabe, T. Taniguchi, T. Amand, and X. Marie, Physical Review Letters **126**, 067403 (2021).
[25] A. V. Stier, K. M. McCreary, B. T. Jonker, J. Kono, and S. A. Crooker, Nature Communications **7**, 10643 (2016).
